# Mesoscopy: Innovations in high-resolution and large-field imaging

**DOI:** 10.1016/j.xinn.2025.100879

**Published:** 2025-03-13

**Authors:** Xin Xu, Jixiang Wang, Qin Luo, Yahui Song, Yi He, Jing Lu, Guohua Shi

**Affiliations:** 1 School of Biomedical Engineering (Suzhou), Division of Life Sciences and Medicine, University of Science and Technology of China, Hefei 230026, China; 2 Suzhou Institute of Biomedical Engineering and Technology, Chinese Academy of Science, Suzhou 215163, China; 3 Hangzhou Institute of Medicine, Chinese Academy of Science, Hangzhou 310002, China

## Main text

**Figure 1:**
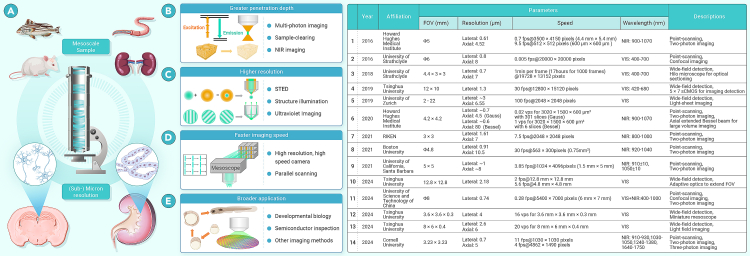
The concept and perspectives of mesoscopy (A) Mesoscopes can image mesoscale samples at (sub-)micron resolution. (B–E) Perspectives on future developments of mesoscopy.

Mesoscopy refers to imaging methodologies that provide a field of view (FOV) ranging from several millimeters to centimeters while achieving cellular or even subcellular resolution (Figure 1). This technological framework employs specially designed large-scale objective lenses to correct aberrations across extended FOVs, synchronized with light-field acquisition modalities through either scanning point detection or large-format array detection. Conventional microscopes, constrained by the limitations of objective lenses, exhibit a trade-off between the FOV and resolution. To achieve both high resolution and a large FOV, common approaches such as FOV stitching and Fourier ptychography were employed. However, these methods were extremely slow and imposed numerous constraints on samples. In 2016, a mesoscopic objective lens was introduced to address these challenges, achieving a 6 mm FOV and 0.7 μm resolution, thereby increasing the imaging throughput of conventional objective lenses by orders of magnitude.^1^ In the same year, this technology was recognized as one of the top ten physics breakthroughs worldwide by *Physics World*. Since then, mesoscopic imaging technology has gradually gained momentum and has been applied in various fields.

## Key components

The objective lens serves as the core component of mesoscopy. The initial design of mesoscopic objective lenses typically involves enlarging traditional objective lens elements. However, this enlargement led to increased aberrations, particularly at the lens periphery. The design of a mesoscopic objective requires the critical aspect of meticulous aberration optimization, such as quasi-aplanatic architectures for dual aberration suppression, curvature-engineered high-order balancing, and freeform/diffractive corrective elements. Cutting-edge mesoscopic objectives show outstanding multimodal abilities. Representative designs achieve broadband performance from 400 to 1,000 nm, with sub-micron resolution over an 8 mm FOV at a numerical aperture (NA) of 0.5. More specialized variants allow three-photon imaging in near-infrared (NIR)-II, with better penetration depth in a 3.5 mm FOV at an NA of 0.75.

As another core component, detectors are functionally categorized into two operational paradigms: point-scanning detectors and wide-field array detectors. (1) Point-scanning detectors are predominantly represented by photomultiplier tubes (PMTs). In most point-scanning mesoscopic imaging systems, conventional PMTs with a sensor diameter of less than 5 mm are used. Nevertheless, some studies have proposed large-target PMT (with a diameter exceeding 10 mm) to collect fluorescence emitted at larger angles. (2) Wide-field array detectors: mesoscopic imaging necessitates large-format sensors with high-pixel densities. Among current commercial cameras, only a few industrial cameras meet this requirement. These cameras are relatively slow, and their parameters, such as noise and sensitivity, are slightly inferior. To improve the imaging effect and speed, some research has pieced together multiple scientific-grade cameras as the detector, but the cost is extremely high. With the development of high-pixel, large-target-surface, scientific-grade cameras, mesoscopic imaging parameters will be enhanced.

## Imaging techniques

Various imaging methods have been developed to address the challenges of mesoscopes, each offering distinct advantages in terms of resolution, speed, and imaging depth. Early mesoscopic scanning systems employed confocal point scanning for single-photon imaging within the visible (VIS) light spectrum (400–700 nm), achieving a 6 mm diameter FOV and a high resolution of 0.8 μm.^1^ Two-photon imaging, due to its superior penetration depth, is essential for *in vivo* brain imaging. Subsequent research has primarily focused on two-photon mesoscopic imaging to investigate the brain. This imaging modality requires NIR pulsed lasers, specifically around 920 nm, to efficiently excite calcium ion fluorescent proteins that indicate neural signal changes. For instance, a mesoscope designed by Yu et al.^2^ captured the signal changes from nearly 6,000 neurons within a 5 × 5 mm FOV at sub-micron resolution. Due to the challenges of chromatic aberration optimization in objective lenses, the design of 920 nm two-photon mesoscopic imaging systems often has a limited spectral range, typically restricted to this specific excitation wavelength. The point-scanning imaging methods, while offering high-resolution and three-dimensional (3D) imaging capabilities, are constrained by slow imaging speeds. A faster mesoscopic imaging approach involves wide-field imaging. The RUSH system, a high-speed mesoscopic representative, uses 35 sCMOS cameras as detectors, achieving a 10 × 12 mm FOV with a resolution of 1.3 μm at 30 fps.^3^ However, this method is not conducive to 3D imaging. Recently, a mesoscope named RUSH3D was proposed, integrating digital adaptive optics and light-field imaging.^4^ This system achieves a resolution of 2.6 × 2.6 × 6 μm and an FOV of 8 × 6 × 0.4 mm, with an impressive imaging speed of 20 volumes per second, marking a 13-fold increase in imaging throughput over the previous RUSH system. Despite the high imaging speed of wide-field methods, they are limited to the visible spectrum and are incompatible with two-photon imaging. Recent research has proposed a mesoscope capable of operating across both visible and NIR spectra, covering wavelengths of 400–1,000 nm. This system enables both single-photon and two-photon imaging, allowing for flexible selection based on sample requirements.^5^ However, this method still relies on point scanning, leading to slow imaging speeds. Achieving a mesoscope that combines high resolution, high penetration depth, and rapid imaging speed remains a significant challenge.

Besides optimizing mesoscopic imaging parameters, other research aims to expand its applications, like downsizing the system and developing advanced image processing algorithms. For system miniaturization, a recent study introduced a 2.1 g miniature mesoscopic imaging system. This system features a 10 mm^2^ FOV with cellular-level resolution, enabling imaging of neurons in freely moving mice. In algorithm research, some studies target imaging quality improvement. For example, light-field mesoscopic imaging algorithms strive to maximize imaging resolution during restoration. Integrating algorithms with deep learning is a future trend, especially in neural signal extraction and analysis. With thousands of neurons in the FOV, quickly locating cells with intensity changes and extracting them is tough. Current deep-learning-based research can extract signals of 14,000 neurons in 17 h.

## Perspectives

We anticipate the future development of mesoscopy from both technological and application perspectives. From a technical standpoint, several potential avenues for advancement can be identified (Figure 1). (1) Greater penetration depth. In *ex vivo* imaging, millimeter-scale depths have been achieved by incorporating sample-clearing techniques. *In vivo* imaging has seen recent advancements with the combination of mesoscopic imaging and three-photon imaging, enabling depths of nearly 1 mm in mouse brain imaging, although this does not span the entire cortical thickness. Future developments may incorporate four-photon imaging, which utilizes even longer wavelengths, to further enhance imaging depth while reducing tissue scattering. (2) Higher resolution. Current mesoscopic imaging systems achieve ∼1 μm resolution. The integration of techniques such as point scanning with stimulated emission depletion and wide-field detection with structured illumination can further enhance resolution. Moreover, the applications of shorter-wavelength light sources, such as deep ultraviolet or extreme ultraviolet illumination, could substantially increase resolution. Illumination with ultraviolet light may increase phototoxicity, so it is generally suitable for imaging *ex vivo* samples. Additionally, some wavelengths, such as 222 nm, cause relatively less damage to organisms and have the potential for *in vivo* imaging. (3) Faster imaging speeds, especially at high depths and high resolution. Current wide-field systems like RUSH and RUSH3D already achieve remarkable speeds, and future advancements in high-pixel, high-frame-rate cameras could further boost performance. However, wide-field detection still has limitations in depth and resolution compared to point scanning. Addressing the challenge of accelerating point scanning could involve transitioning from point to line scanning, capturing multiple points simultaneously while maintaining high resolution and ensuring imaging depth.

In terms of applications, mesoscopic imaging is poised to make significant strides across various domains. (1) Developmental biology. With its high resolution and large FOV, mesoscopic imaging enables the observation of dynamic cell behaviors during development. It can track individual cell migration, morphology, and division cycles while also revealing synchronized activities within cell populations, such as collective migration and cooperation. This aids researchers in understanding how coordinated cellular actions drive organ and tissue formation. (2) Semiconductor inspection. Besides being applied in the life sciences, mesoscopic imaging has potential uses in industrial fields as well. The high-throughput imaging capability of mesoscopy can accelerate the process of semiconductor defect detection. By utilizing fast image acquisition and automated analysis, it can significantly enhance the efficiency of defect screening on production lines. Although mesoscopic imaging technology is still in its early stages, it holds vast potential for future development and applications. (3) Combinations with other imaging methods. Mesoscopic imaging can integrate with modalities like fluorescence lifetime imaging (FLIM) and fluorescence resonance energy transfer (FRET) to expand applications. For instance, combining FLIM with mesoscopic imaging allows for observing fluorescence lifetime distribution in tumor cells within large-scale tissues and analyzing metabolic activity and pathways for cancer research. FRET, detecting intermolecular interactions, can study spatial relationships in a long pathway of relevant molecules on synaptic membranes during neurotransmitter release.

In conclusion, mesoscopic imaging represents a transformative technology with the potential to revolutionize both scientific research and industrial applications. While challenges remain in optimizing speed, resolution, and penetration depth, the continued development of mesoscopic systems promises to open new frontiers in neuroscience, developmental biology, and other areas.

## Funding And Acknowledgments

This work was supported by the Chinese Academy of Sciences Project for Young Scientists in Basic Research (YSBR067), the 10.13039/501100004608Natural Science Foundation of Jiangsu Province (BK20240024), and the 10.13039/501100004739Youth Innovation Promotion Association of the Chinese Academy of Sciences (Y2023087). The funders had no role in the study design, data collection and analysis, decision to publish, or preparation of the manuscript.

## Declaration of interests

The authors declare no competing interests.
